# Endotoxin and CD14 in the progression of biliary atresia

**DOI:** 10.1186/1479-5876-8-138

**Published:** 2010-12-21

**Authors:** Ming-Huei Chou, Jiin-Haur Chuang, Hock-Liew Eng, Ching-Mei Chen, Chiou-Huey Wang, Chao-Long Chen, Tsun-Mei Lin

**Affiliations:** 1 Institute of Basic Medical Sciences, National Chang Kung University, Tainan, Taiwan; 2 Graduate Institute of Clinical Medical Sciences, Chang Gung University, Kaohsiung, Taiwan; 3 Department of Surgery, Chang Gung Memorial Hospital - Kaohsiung Medical Center, Chang Gung University College of Medicine, Kaohsiung, Taiwan; 4 Department of Pathology, Chang Gung Memorial Hospital - Kaohsiung Medical Center, Chang Gung University College of Medicine, Kaohsiung, Taiwan; 5 Department of Laboratory Medicine, E-DA Hospital/I-SHOU University, Kaohsiung, Taiwan; 6 Department of Medical Research, E-DA Hospital/I-SHOU University, Kaohsiung, Taiwan

## Abstract

**Background:**

Biliary atresia (BA) is a typical cholestatic neonatal disease, characterized by obliteration of intra- and/or extra-hepatic bile ducts. However, the mechanisms contributing to the pathogenesis of BA remain uncertain. Because of decreased bile flow, infectious complications and damaging endotoxemia occur frequently in patients with BA. The aim of this study was to investigate endotoxin levels in patients with BA and the relation of these levels with the expression of the endotoxin receptor, CD14.

**Methods:**

The plasma levels of endotoxin and soluble CD14 were measured with a pyrochrome Limulus amebocyte lysate assay and enzyme-linked immunosorbent assay in patients with early-stage BA when they received the Kasai procedure (KP), in patients who were jaundice-free post-KP and followed-up at the outpatient department, in patients with late-stage BA when they received liver transplantation, and in patients with choledochal cysts. The correlation of CD14 expression with endotoxin levels in rats following common bile duct ligation was investigated.

**Results:**

The results demonstrated a significantly higher hepatic CD14 mRNA and soluble CD14 plasma levels in patients with early-stage BA relative to those with late-stage BA. However, plasma endotoxin levels were significantly higher in both the early and late stages of BA relative to controls. In rat model, the results demonstrated that both endotoxin and CD14 levels were significantly increased in liver tissues of rats following bile duct ligation.

**Conclusions:**

The significant increase in plasma endotoxin and soluble CD14 levels during BA implies a possible involvement of endotoxin stimulated CD14 production by hepatocytes in the early stage of BA for removal of endotoxin; whereas, endotoxin signaling likely induced liver injury and impaired soluble CD14 synthesis in the late stages of BA.

## Background

Biliary atresia (BA) is a typical cholestatic neonatal disease, characterized by obliteration of intra- and/or extra-hepatic bile ducts with repeated episodes of cholangitis and progressive liver fibrosis and cirrhosis [1-3]. However, the mechanisms contributing to the pathogenesis of BA remain uncertain. A decrease of bile flow to the bowel may promote bacterial translocation to the liver and increase endotoxin or lipopolysaccharide (LPS) levels in the peripheral circulation [4]. LPS represent the major component of the outer membrane of Gram-negative bacteria and has been implicated in sepsis, organ failure, and shock [5]. In experimental studies on healthy animals, LPS is cleared from the circulation within a few minutes of intravenous injection, and the majority of LPS is traced to the liver [6,7]. In addition to clearing LPS, the liver also responds to the presence of LPS with production of cytokines and reactive oxygen intermediates. Accumulating evidence suggests that both endotoxins and pro-inflammatory cytokines participate in liver damage during endotoxemia [8,9].

CD14 is a glycosylphosphatidylinositol-anchored LPS receptor. It was first reported as a differentiation marker expressed on the surface of macrophages, neutrophils, and other myeloid lineage cells [10-13]. Human hepatocytes demonstrate production of CD14 similar to that of an acute phase protein [14]. However, there is limited information on the proportional change of CD14 in the liver and the consequent pathogenetic effects on LPS-induced liver injury. Although increased expression of CD14 in surgically biopsied specimens of BA have been reported, the exact mechanism of such over-expression of CD14 is yet to be elucidated [15]. Our previous investigation revealed that the single nucleotide polymorphism at CD14/-159 is associated with the development BA and idiopathic neonatal cholestasis [16]. How the liver responds to LPS-induced injury is virtually unknown at present [17,18]. Kupffer cells and sinusoidal endothelial cells express the membrane form of CD14 (mCD14) in the liver [19,20], while hepatocytes are the main producers of soluble CD14 (sCD14) [21,22]. However, the proportional change of CD14 production in the liver and the subsequent effects on LPS-induced liver injury during BA is not clear.

In this study, we investigated the role of CD14 in BA-associated liver injury, with particular emphasis on the correlation between CD14 expression and endotoxin levels in the liver tissue and plasma of patients in the early and late stages of BA. We further elucidated the expression and regulation of CD14 in a rat model following bile duct ligation (BDL).

## Methods

### Patients and samples

Liver biopsy specimens were obtained from nine patients with early-stage BA (four males and five females) during Kasai's procedure (KP), from nine patients with late-stage BA (four males and five females) during liver transplantation for failed KP, and from nine patients with choledochal cysts (CCs) during surgical correction (2 male and 7 female). Control liver biopsy samples were obtained from five children with neonatal hepatitis and two who had focal hepatoblastoma. Plasma samples were obtained from 41 patients with early-stage BA, 25 patients post-KP who were jaundice-free and were followed-up at the outpatient department (OPD), 49 patients with late-stage BA, 9 patients with CC, and 7 healthy young infants. All of the liver and blood samples were immediately frozen at -80°C for later laboratory tests. The clinical characteristics and detailed history of the patients, including the age when the patient underwent the procedure, sex, serum aspartate aminotransferase (AST) levels, and total bilirubin are summarized in Table 1. Informed consent was obtained from the patients or their legal guardians, and the experiments were approved by the Ethics & Clinical Trial Committee of the Chang Gung Memorial Hospital, Taiwan.

**Table 1:** Clinical characteristics of the child patients for this study

	Con-C	Early stage of BA		OPD	Late stage of BA		CC
	
	Plasma	Plasma	Liver	Plasma	Plasma	Liver	Plasma & Liver
**Sample No**	7	41	9	25	49	9	9
**Age (months)**	18 ± 24	2.4 ± 1.2	2 ± 1	24 ± 16	15 ± 10	15 ± 6	22 ± 14
**Sex (M/F)**	4/3	23/16	4/5	7/18	19/21	4/5	2/7
**AST (U/l)**	ND	200 ± 175^†^	181 ± 130	ND	276 ± 241	246 ± 114	298 ± 228
**T. Bil (mg/dl)**	ND	9.0 ± 2.9^†^	9.4 ± 3.0	ND	16 ± 1.0	18 ± 10	6.5 ± 6.0^†^
**D. Bil (mg/dl)**	ND	6.3 ± 2.0^†^	7.0 ± 2.3^†^	ND	12 ± 7.5	13 ± 7.2	5.6 ± 4.0^†^
**sCD14 (μg/ml)**	4.0 ± 0.8	4.7 ± 1.7^†^	47 ± 14	4.2 ± 1.4^†^	2.7 ± 1.5*^‡^	49 ± 12	4.0 ± 2.0^†^
**Endotoxin (EU/ml)**	2.0 ± 1.0	6.2 ± 5.0*^‡^	17 ± 4.0	2.2 ± 5.0^†^	6.7 ± 5.0*^‡^	16 ± 7.0	6.5 ± 4.0*^‡^

Con-C, control-children; Early stage of BA, Kasai's procedure for biliary atresia; OPD, Jaundice-free post-Kasai BA patients followed at the outpatient department; Late stage of BA, liver transplantation for biliary atresia; CC, choledochal cyst; AST, alanine aminotransferase; D. Bil, direct bilirubin; T. Bil, total bilirubin; ND, non detection; sCD14, souble CD14; y, years. Value are expressed as the mean ± SD. *P < 0.05 *vs *control; ^†^*P *< 0.05 *vs *late stage of BA; ^‡^*P *< 0.05 *vs *OPD.

### Animals

Male Sprague-Dawley rats weighting 300-330 g and about 8 weeks old were divided into three groups: the BDL group (n = 48) received a common bile duct complete double ligation, the sham group received a sham operation (n = 48), and the normal control group (n = 6). All animal experiments were performed in accordance with and approved by the Animal Care and Use Committee of Chang Gung Memorial Hospital at Kaohsiung. Blood samples were collected at time of sacrifice (3 hrs, 6 hrs, 12 hrs, 24 hrs, 3 days, 7 days, 14 days, and 21 days), and six rats were included in each subgroup. Serum enzymes and bilirubin levels were determined using a biochemistry auto-analyzer (Model 7450; Hitachi, Tokyo, Japan). Liver tissues were either snap frozen and homogenized in T-PER tissue protein extraction reagent (Pierce Chemical, Rockford, IL) for protein determination or fixed in 4% paraformaldehyde and embedded in paraffin for immunohistochemical analysis.

### Determination of sCD14 levels by ELISA

The sCD14 levels of plasma were determined using a commercially available enzyme-linked immunosorbent assay (ELISA; R & D Systems, Minneapolis, MN) according to the manufacture's instructions. Samples were diluted 1:200 and analyzed, and each sample was measured in duplicate.

### *Limulus *amebocyte lysate (LAL) test

Plasma specimens were collected aseptically in nonpyrogenic containers. The plasma and liver specimens were diluted 1:10 and assayed for endotoxin with a commercially available pyrochrome LAL kit (Associates of Cape Cod, Falmouth, MA) according to the manufacture's instructions.

### Real-time quantitative reverse transcription-polymerase chain reaction (qRT-PCR)

Frozen liver samples (0.1 g/per sample) were homogenized, and total RNA was extracted using TRIzol (Invitrogen, Carlsbad, CA). The RNA isolates were quantified at A_260/280 _ratio of 1.7-2.0. A total of 2 μg of RNA was added to 0.1 μg of oligo-d (T)_15 _following the protocol for SuperScripIIRT (Invitrogen, Carlsbad, CA). Quantitative PCR was performed in a final volume of 20-μl SYBR Green PCR mixture (Applied Biosystems, Foster City, CA), and each sample was analyzed in duplicate. Each reaction mixture contained 0.2 pmole/ul of each primer, 1× SYBR Green PCR Master Mix, and 1-5 ng of cDNA. Thermal cycling was initiated with a 2 min incubation at 50°C, followed by a denaturation step of 10 min at 95°C, and then 40 cycles of PCR consisting of 95°C for 15 seconds, 60°C for 20 seconds, and 72°C for 30 seconds. β-actin was used as an internal control for analyzing CD14 mRNA levels. The sequence of the PCR primers were designed based on cDNA sequences from Genbank as follows: CD14 forward primer 5'-TAT GCT GACACG GTC AAG GC-3', CD14 reverse primer 5'-ATT GTC AGA CAG GTC TAG GC-3', β-actin forward primer 5'-TCA CCC ACA ATG TGC CCA TCT TCG A-3', and β-actin reverse primer 5'-CAG CGG AAC CGC TCA TTG CCA ATG G-3'.

The quantification of the CD14 mRNA was achieved with an ABI PRISM 7700 Sequence Detection System (Applied Biosystems, Warrington, WA) using comparative methods. Ct values of CD14 were normalized to the Ct value of a housekeeping gene (β-actin).

### Immunohistochemical staining for CD14 and lipid A

Immunoreactive CD14 and lipid-A staining was performed on paraffin-embedded, formalin fixed, archival human liver tissues obtained from the Department of Pathology, Kaohsiung Chang Gung Memorial Hospital, Taiwan. In the animal study, formalin-fixed, paraffin-embedded liver tissues were used. Two-micrometer sections were deparaffinized, treated with 3% hydrogen peroxide to inactivate the endogenous peroxidase activity, and microwaved for 7 min in 10-mM citrate buffer (pH 6.0) to retrieve the antigen. The sections were then incubated in PBS supplemented with 5% fetal calf serum for 10 min to block background interactions. The sections were then incubated with a rabbit anti-CD14 antibody (Santa Cruz Biotechnology, Santa Cruz, CA) or a mouse anti-lipid A antibody (HyCult Biotechnology, The Netherlands) at 37°C for 2 hrs. The sections were washed with PBS supplemented with 0.05% Tween 20 and then incubated for 10 min with the secondary antibodies (SuperPicture; Zymed Laboratories, Francisco, CA). DAB color substrate (DAKO, Carpinteria, CA) was added to cover each section, and the reaction was stopped with ddH_2_O. The slides were counterstained with hematoxylin, and mounted in mounting medium.

### In situ hybridization

In situ hybridization was performed essentially as described by Wilkinson[23]. The riboprobe was generated from a pGEM-T vector containing a 250 bp cDNA sequence of *CD14 *and labeled with DIG-11-UTP by *in vitro *transcription with SP6 and T7 RNA polymerase, followed by DIG RNA labeling (Roche Applied Science, Germany). Liver tissues were treated according to the protocol for immunohistochemical analysis with deparaffinization, rehydration, removal of endogenous peroxidase activity, and antigen retrieval. Sections were digested with 20 μg/ml of proteinase K solution at 37°C for 25 min and then prehybridized with 5× SSC buffer. A total of 50-μl hybridization mixture containing denatured RNA probes was used and hybridized with the sections at 55°C overnight. After hybridization, the sections were treated with 20 μg/ml of RNase solution at 37°C for 30 min to remove free RNA probes and then washed with 1× SSC buffer for 5 min and 0.2× SSC containing 0.01% SDS in a 55°C water bath for 15 min. Sections were blocked in PBS supplemented with 5% FBS and incubated with an anti-digoxigenin antibody conjugated with horseradish peroxidase (diluted 1:1000, containing 2% FCS) in blocking buffer for 2 h at room temperature. The sections were washed with PBS supplemented with 0.05% Tween 20 and then DAB color substrate (DAKO, Carpinteria, CA) was added to cover each section, and the reaction was stopped with ddH_2_O. The slides were counterstained with hematoxylin, and mounted in mounting medium.

### Statistics analysis

Data are presented as the mean ± standard deviation (SD). The distributions of paired measurements were compared using the nonparametric Wilcoxon matched-pairs test. The Mann-Whitney test and Wilcoxon signed-ranks test (nonparametric) were used to evaluate the statistical significance of the results using the SPSS-16 software package (SPSS, Chicago, USA). A *P *value of less than 0.05 was considered significant.

## Results

### Plasma CD14 and endotoxin levels in patients with BA

**Figure 1:**
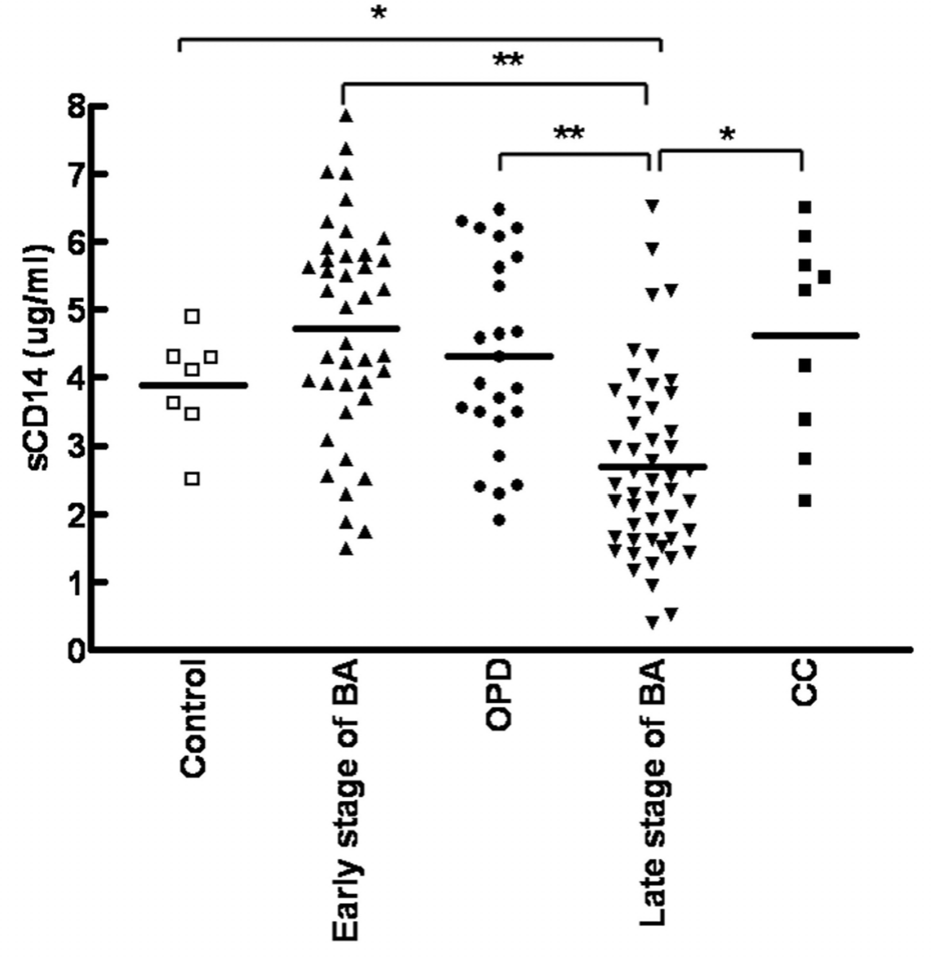
**Plasma sCD14 levels in patients with BA**. Quantitative analysis of soluble CD14 by ELISA in the plasma of 41 patients with early-stage biliary atresia (BA), 25 patients followed-up at the outpatient department (OPD) post-Kasai, 49 patients with late-stage BA, 9 patients with choledochal cysts (CC), and 7 healthy controls. Data represent the mean ± SD from duplicate experiments. Statistical differences were tested by nonparametric Wilcoxon matched-pairs test. *p <0.05 and **p <0.01 *vs*. late-stage BA.

Plasma sCD14 levels were analyzed by ELISA and found to be significantly higher in patients with early-stage BA (4696 ± 1652 ng/ml), patients with BA who were jaundice-free and followed up at the OPD (4308 ± 1428 ng/ml), and patients with CCs (4393 ± 1900 ng/ml) relative to patients with late-stage BA (2722 ± 1453 ng/ml, *P <*0.001). Although sCD14 levels in the early-stage BA, OPD, and CC groups were higher than controls (3879 ± 767 ng/ml), these differences were not statistically significant (*P *= 0.134, *P *= 0.447, and *P *= 0.442, respectively) (Figure 1). There were seven patients with BA whose plasma samples were available for both the early and late stages. For these patients, plasma sCD14 levels were significantly higher in the early stage (4445 ± 237 ng/ml) compared to those in the late stage (2183 ± 153 ng/ml) based on paired t-test analysis (*P *< 0.001).

**Figure 2:**
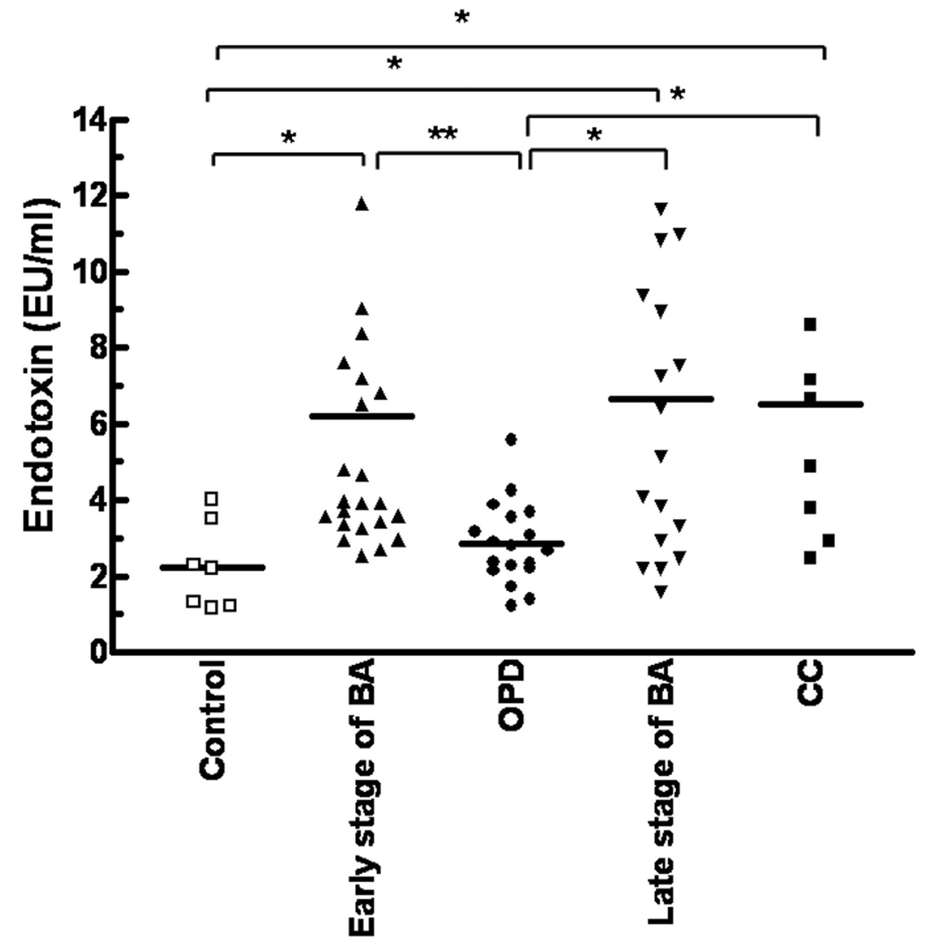
**Plasma endotoxin levels in patients with BA**. Detection of plasma endotoxin levels by chromogenic *Limulus *amebocyte lysate (LAL) test in 7 healthy controls, 24 patients with early-stage BA, 18 patients followed-up at the OPD post-Kasai, 18 patients with late-stage BA, and 9 patients with CC. Data represent the mean ± SD of duplicate experiments. Statistical differences were tested by nonparametric Wilcoxon matched-pairs test. *p < 0.05, **p < 0.01.

There was no significant difference in endotoxin levels between the patients with early-stage BA (6.18 ± 4.59 EU/ml) and those with late-stage BA (6.6 ± 4.58 EU/ml, *P *= 0.74) However, the levels of plasma endotoxin in patients in either stage of BA and in patients with CC (6.51 ± 4.27 EU/ml) were significantly higher than controls (2.2 ± 1.1 EU/ml, *P *< 0.001). The plasma endotoxin levels in the patients with BA that were jaundice-free and followed up in the OPD (2.8 ± 1 EU/ml) were markedly lower than those patients in either stage of BA and those with CC (*P *< 0.001) (Figure 2).

### CD14 mRNA and protein expression in liver tissues of patients with BA

**Figure 3:**
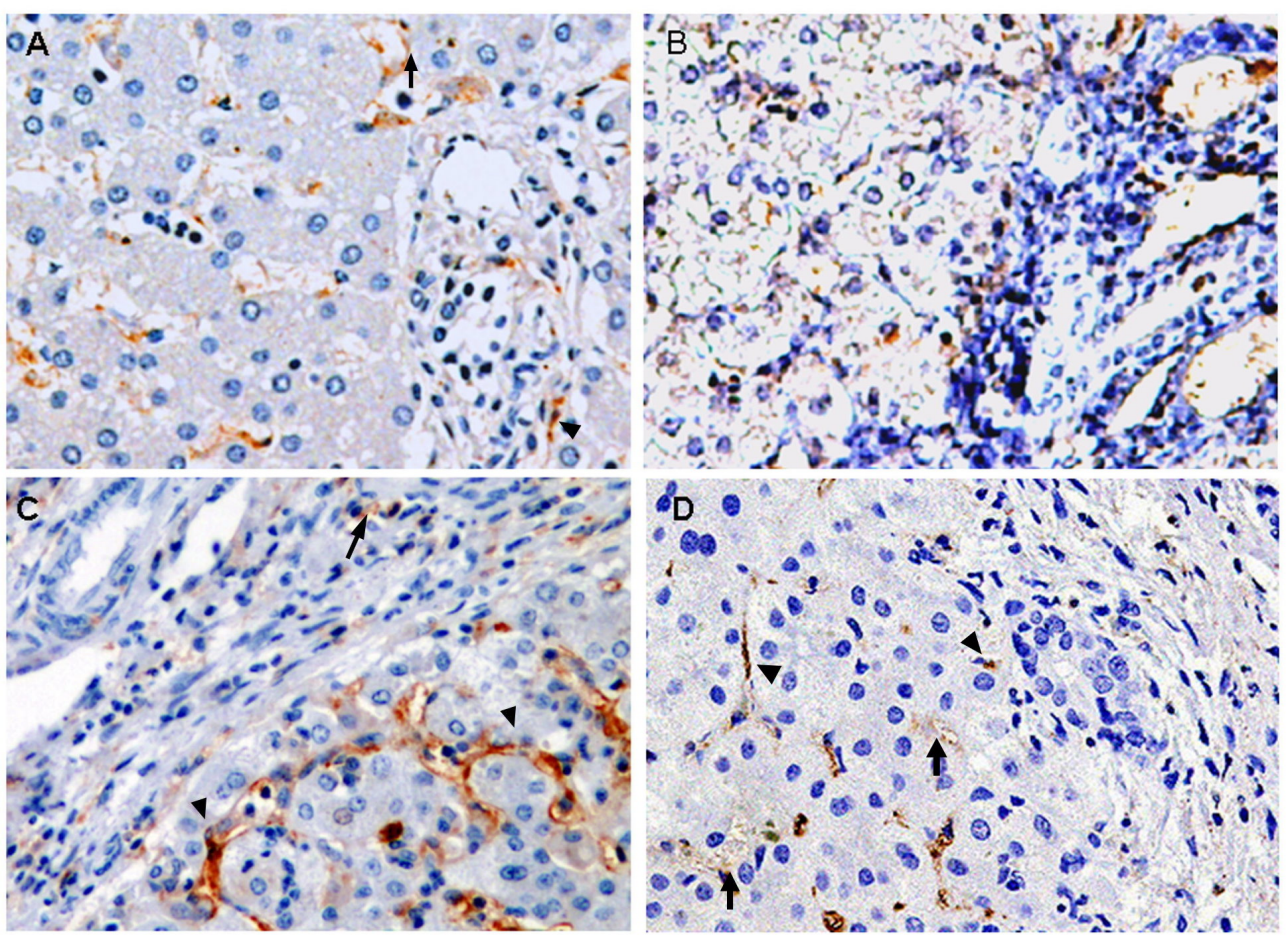
**CD14 expression in liver tissues of patients with BA**. Comparison of CD14 expression in paraffin-embedded liver tissue sections among the control group (biopsy from neonatal hepatitis and hepatoblastoma) (A), patients with CC (B), patients with early-stage BA (C), and patients with late-stage BA (D). Liver sections were stained with a monoclonal antibody against CD14 (dark brown) and counterstained with hematoxylin.. Kupffer cells (arrow) and sinusoidal endothelial cells (arrowhead) showed positive immunostain for CD14. Original magnification: × 200

**Figure 4:**
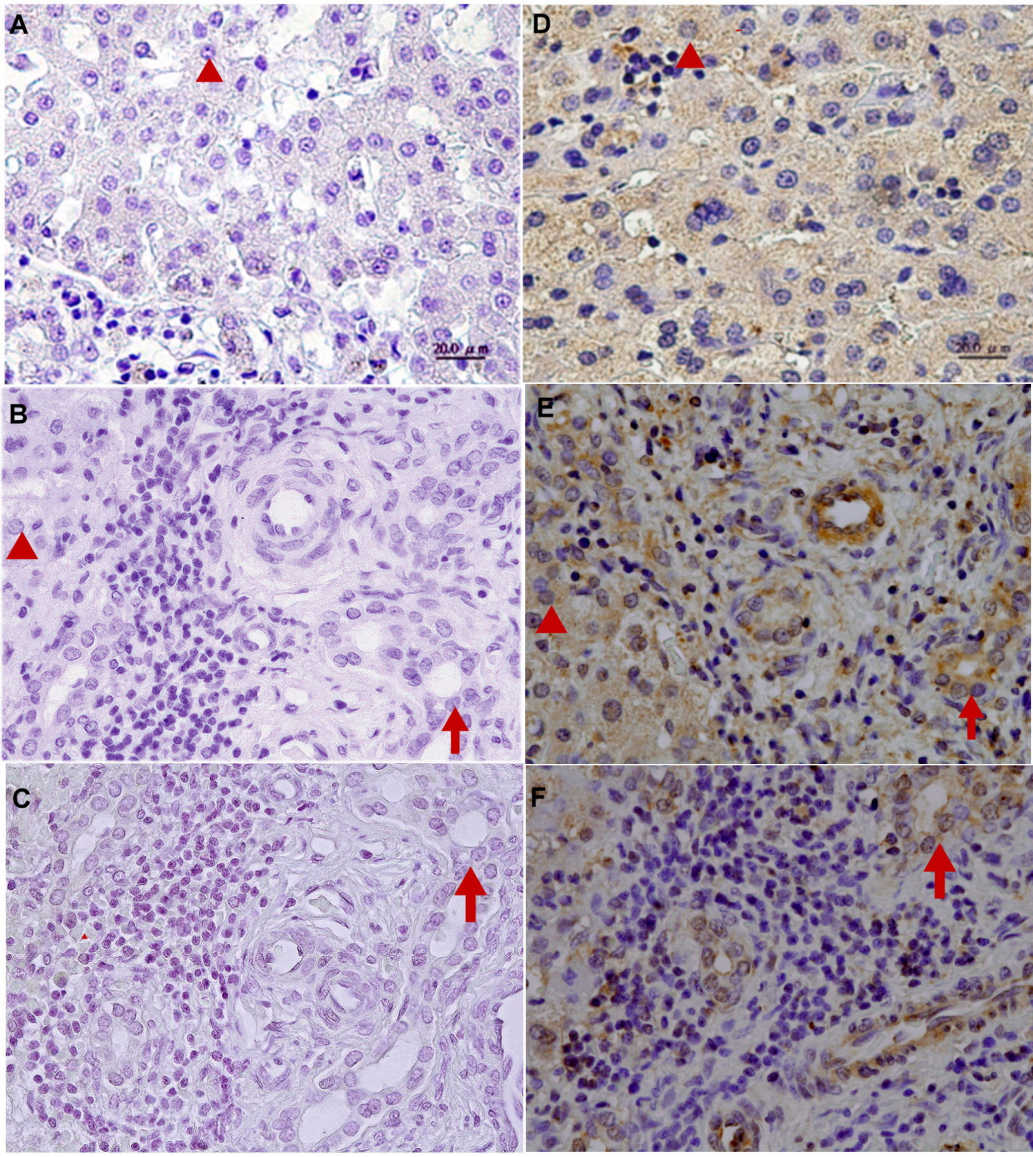
**CD14 mRNA expression in liver tissues of patients with BA**. *In situ *hybridization of CD14 mRNA in the livers of patients with early- and late-stage BA. CD14 is stained brown by *in situ *hybridization with a DIG-labeled CD14 sense (A-C) and antisense RNA probe (D-F). The paraffin-embedded sections from patients with hepatoblastoma as control (A, D), early-stage BA (B, E) and late-stage BA (C, F) Tissues were counterstained with hematoxylin. The CD14 mRNA expression pattern mainly localized in the hepatocytes (arrowhead) and the bile duct epithelial cells (arrow). Original magnification: × 200.

Paraffin-embedded liver sections from two control patients, five patients with early-stage BA, and five patients with late-stage BA were analyzed for CD14 localization by immunohistochemical staining. In all tissues, CD14 was observed in the parenchyma of the hepatic lobules, where Kupffer cells and sinusoidal endothelial cells were immunostained positive and the arterial and venous endothelium, bile duct epithelial cells, and hepatocytes were negative. However, in CC (Figure 3B) and early-stage BA tissues (Figure 3C), a clear and more intense CD14 staining was observed in Kupffer cells and sinusoidal endothelial cells. The intensity of CD14 expression was significantly higher in the early-stage BA tissues (Figure 3C) compared to the late-stage BA and control tissues (Figure 3D and 3A). To further ensure proper identification of the cell types expressing CD14 in the liver tissues, *in situ *hybridization of the CD14 mRNA was performed with a DIG-labeled CD14 sense (Figure 4A-C) and antisense RNA probe (Figure 4D-F). In addition to Kupffer cells and sinusoidal endothelial cells, hepatocytes and bile duct cells were also demonstrated positive for CD14 mRNA in the parenchyma of the hepatic lobules in control tissues (Figure 4A and 4D). However, in the early-stage BA tissues (Figure 4B and 4E), the CD14 mRNA presented a constitutive and uniform expression pattern mainly localized in the hepatocytes and the bile duct epithelial cells (Figure 4E). The expression of the CD14 mRNA was higher in the early-stage BA tissues (Figure 4E) than that of control tissues (Figure 4D), but its expression was significantly decreased in the late-stage BA tissues due to loss of hepatocytes (Figure 4F). In addition, on qRT-PCR analysis, CD14 mRNA levels were 5-fold higher in early-stage BA tissues (n = 9) relative to the late-stage BA tissues (n = 9) (6.7 ± 1.2 *vs*. 1.4 ± 0.6, *P *= 0.002).

### The localization of endotoxin in the liver tissues

**Figure 5:**
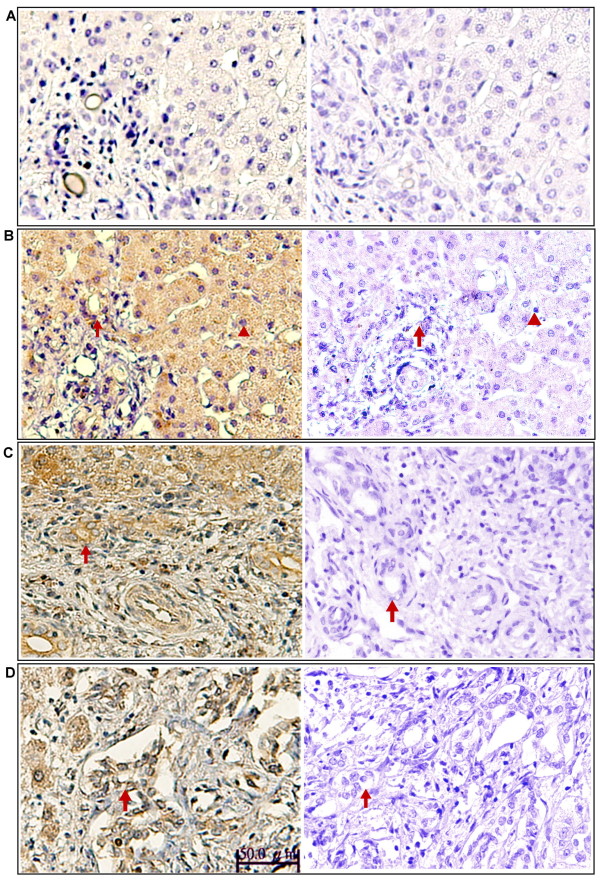
**Endotoxin levels in liver tissues of patients with BA**. Immunohistochemical staining for endotoxin in the liver tissues of controls (biopsy from neonatal hepatitis and hepatoplastoma) (A), patients with CC (B), patients with early-stage BA (C), and patients with late-stage BA (D). Liver sections were stained using a monoclonal antibody against lipid A (HM2046) (left column), mouse IgG1 isotype control antibody (ab27479) (right column) and counterstained with hematoxylin. Lipid-A immunoreactivity was detected in hepatocytes (arrowhead) and biliary epithelial cells (arrow), Original magnification: × 200.

Immunohistochemical staining using a monoclonal antibody against lipid A was performed in liver tissue sections for detecting the localization of endotoxin. In the normal liver tissues (Figure 5A), immunoreactivity to lipid A was weak or absent. However, lipid-A immunoreactivity was strongly detected around the portal area in hepatocytes, Kupffer cells, biliary epithelial cells, and some infiltrating cells in patients with CC (Figure 5B) and in patients with early-stage BA (Figure 5C). In patients with late-stage BA, immunoreactivity to lipid A was detected around sites of fibrous septum formation in hepatic parenchymal cells, Kupffer cells, and biliary epithelial cells (Figure 5D). In the liver of patients with BA, both hepatocytes and nonparenchymal liver cells, such as biliary epithelial cells and Kupffer cells, demonstrated evident uptake of endotoxin, paralleling the high circulating plasma levels of endotoxin.

### Serum enzymes and bilirubin levels in BDL rat model

**Figure 6:**
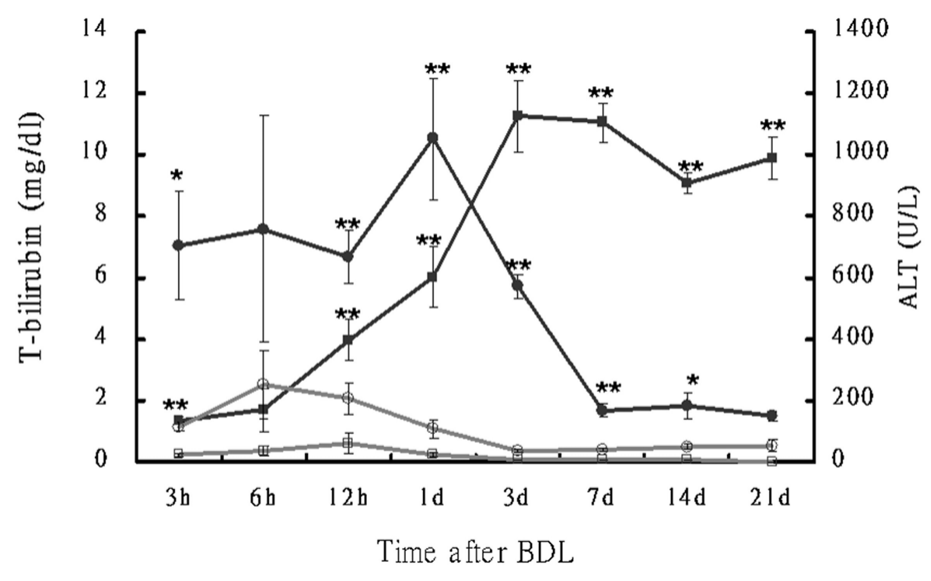
**Total bilirubin and ALT levels in rats**. Time course of total bilirubin (T-bilirubin; square) and alanine transaminase (ALT; circle) in rat plasma after bile duct ligation (BDL; closed symbols) or sham (open symbols) operation. Blood samples were collected at the time points indicated. T-bilirubin and ALT were assayed using a biochemistry auto-analyzer (Model 7450; Hitachi, Tokyo, Japan). Values are mean ± SD (n = 6 in each subgroup). *p < 0.05, **p < 0.005 (sham *vs*. BDL groups).

**Figure 7:**
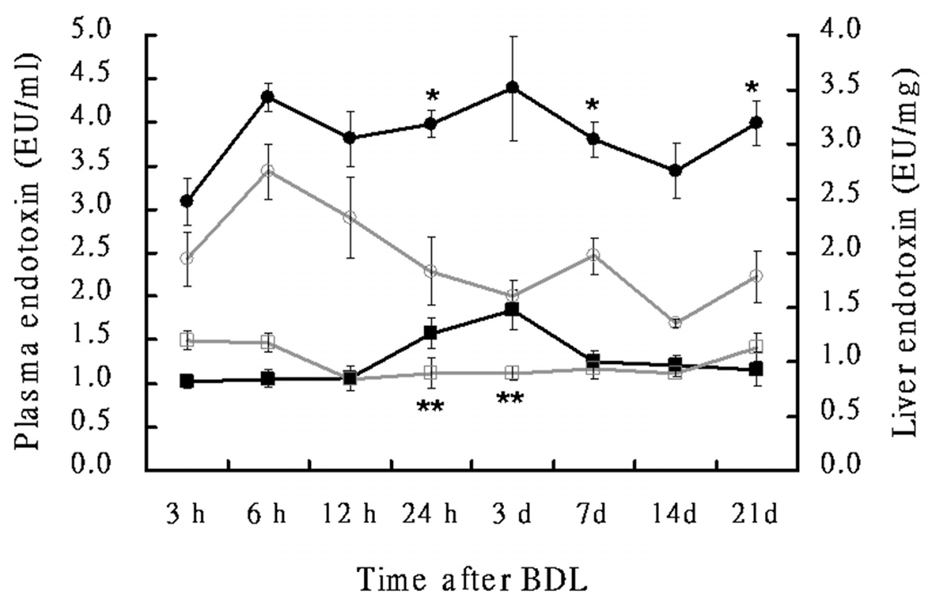
**Endotoxin levels of plasma and liver tissues in rats**. Time course of endotoxin levels in the liver (circles) and plasma (squares) after BDL (closed symbols) or sham (open symbols) operation. Blood samples were collected at the time points indicated. Endotoxin was assayed using a pyrochrome LAL kit (Associates of Cape Cod, Falmouth, MA). Values are mean ± SD (n = 6 in each subgroup). *p <0.05, **p < 0.005 (sham *vs*. BDL groups).

In the BDL rat model, hepatic injury was associated with an increase in serum alanine aminotransferase (ALT) and bilirubin levels. As shown in Figure 6, ALT increased to 1053 IU/L (BDL vs. sham; *P *= 0.001) at Day 1 after ligation, indicating severe liver injury after BDL. ALT levels decreased afterward and reached a new steady-state level of about 180 U/L after Day 7 post-ligation. However, the total bilirubin continuously increased after ligation and reached its peak at Day 3 (BDL vs. sham; 11.26 ± 1.18 vs. 0.1 ± 0 mg/dL, *P *< 0.001) and remained high level throughout the BDL period. The endotoxin levels in the plasma and liver tissues were also significantly increased after Day 1 post-ligation and paralleled an increase in plasma bilirubin levels (Figure 7).

### CD14 and lipid-A detection in the BLD rat model

**Figure 8:**
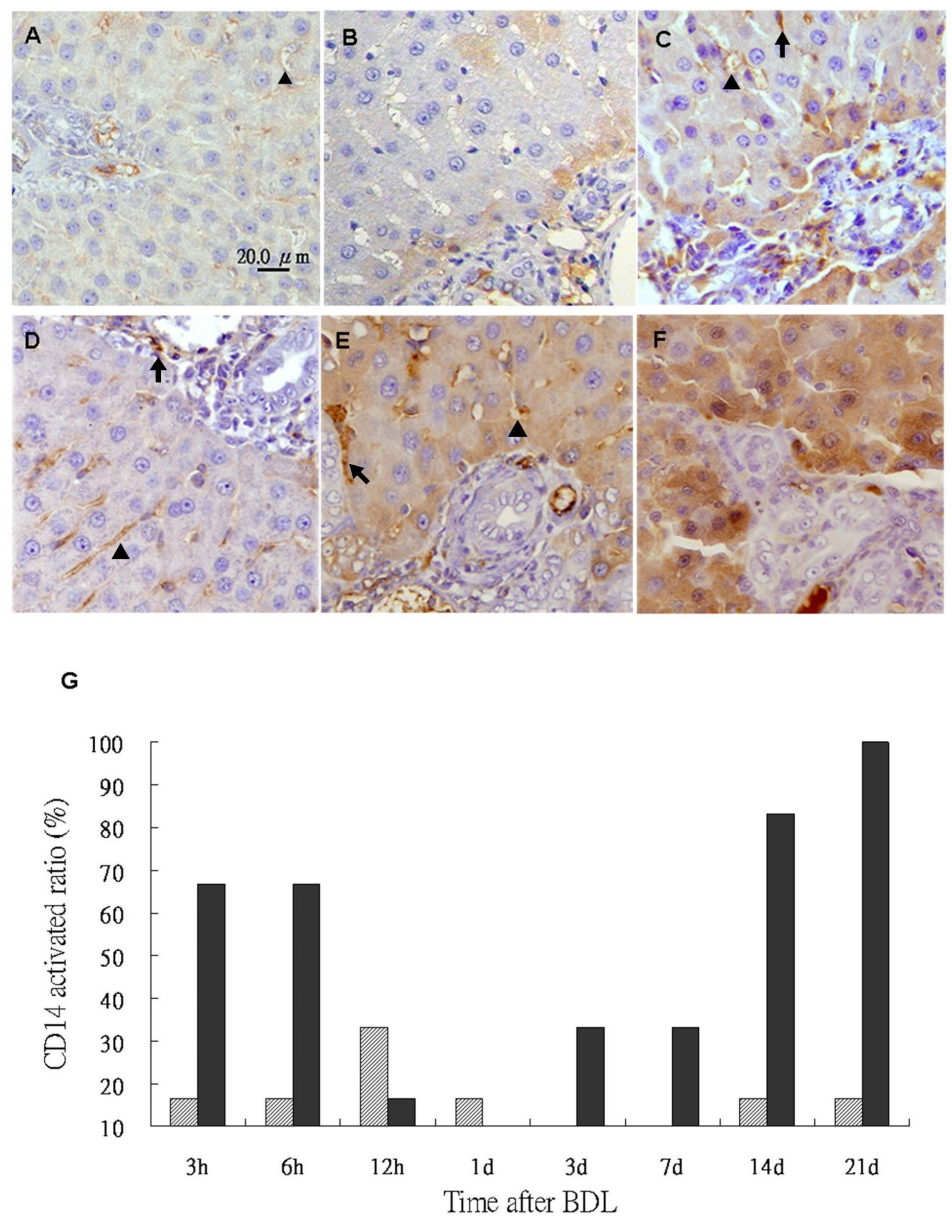
**CD14 expression in the liver tissues of rats**. CD14 staining in the liver tissues of rats from the sham and BDL groups. Staining of liver sections using a polyclonal antibody against CD14 shows negligible or no staining in any liver cells in the control (A). Positive staining in the Kupffer cells (arrow), the sinusoidal endothelial cells (arrowhead) and more strongly in hepatocytes around the portal zones at 3 h after sham-operation (B), and at 3 h, 1 d, 1w, 3w (C-F) after BDL. Tissues were counterstained with hematoxylin. Original magnification: × 200. The ratio of CD14 activated (CD14 positive cells were present in over 10% of the tissue area) of the sham (pale bar) and BDL (black bar) groups (G).

**Figure 9:**
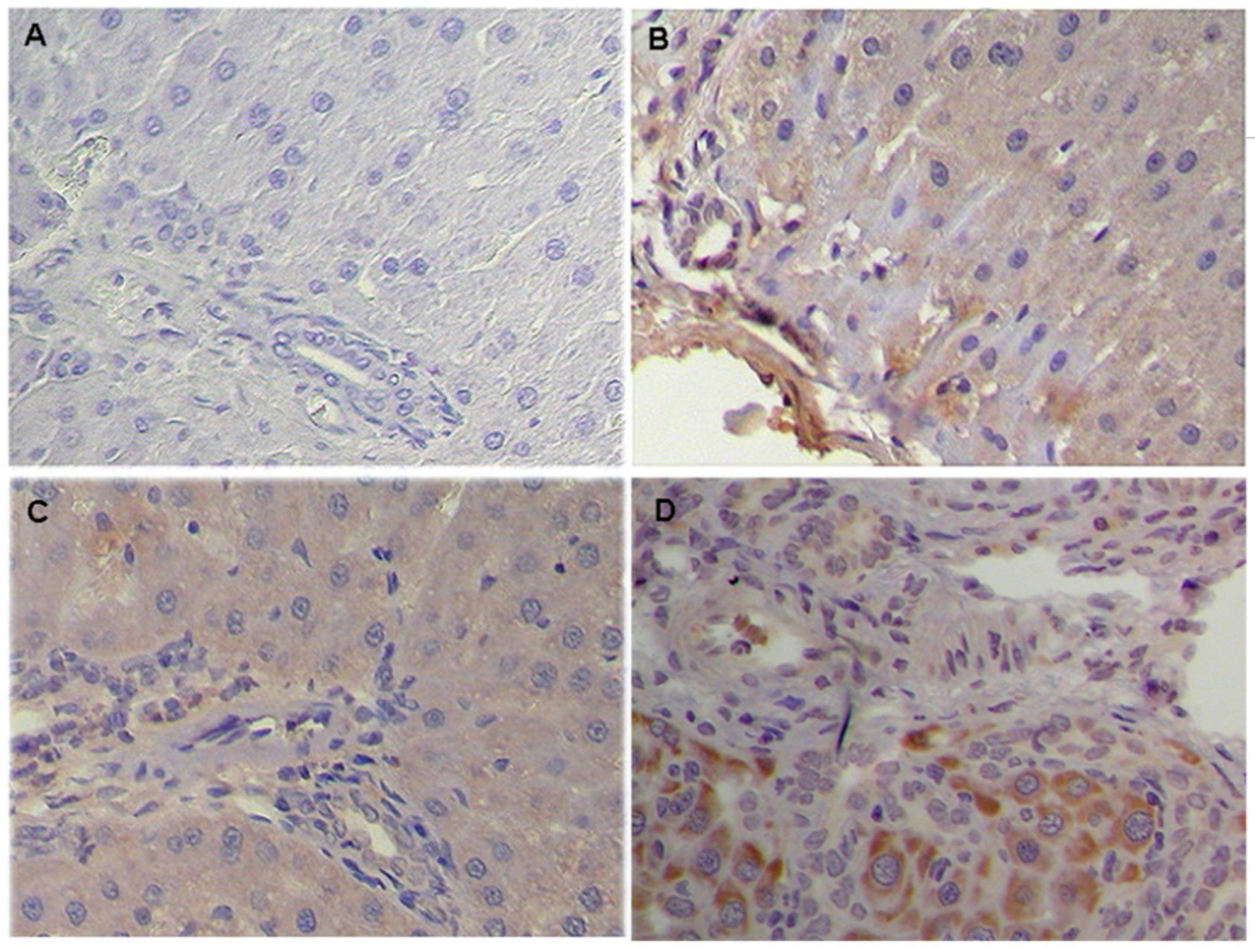
**CD14 mRNA expression in the liver tissues of rats**. *In situ *hybridization of CD14 mRNA in the livers from sham operated and BDL groups. CD14 is stained brown by *in situ *hybridization with a DIG-labeled CD14 antisense RNA probe. The paraffin-embedded sections were hybridized with a sense RNA probe against CD14 in normal tissues as a negative control (A). CD14 is expressed throughout the parenchyma of the liver tissues of normal controls (B), sham-operated (C) and BDL (D) for 14 days. Tissues were counterstained with hematoxylin. Original magnification: × 200.

Temporal expression of CD14 in hepatocytes was assessed via immunohistochemical analysis in rats. CD14 was expressed in the Kupffer cells, sinusoid endothelial cells and more strongly in hepatocytes around the portal zones (Figure 8B-F) in rat liver tissues. A significantly higher CD14 expression was discerned in hepatocytes of BDL rats (Figure 8C-F) as compared to the sham-operated group. Quantitative evaluation of CD14 positive cells in live tissues was performed by an experienced hepatopathologist. If CD14 positive cells were present in over 10% of the tissue area, CD14 was considered activated. As shown in Table 2, CD14 activation was a dynamic phenomenon in BDL group. The expression of CD14 in hepatocytes was enhanced at 3-6 h post-ligation and returned to baseline levels by 24 h. Then, CD14 expression was demonstrated to increase again after 7 days. The BDL rats also shown a significantly higher CD14 activation in hepatocytes compared to the sham-operated group (Figure 8G). I*n situ *hybridization of mRNA of CD14 was performed in rat liver tissues. In addition to Kupffer cells and sinusoidal endothelial cells, CD14 mRNA was demonstrated in hepatocytes and bile duct cells of the hepatic lobules in control tissues (Figure 9A and 9B). The expression of CD14 mRNA in liver tissue of BDL rats was higher than that of the sham-operated group at day 14 after BDL, especially in the hepatocytes (Figure 9D and 9C).

**Table 2:** Indexes e of rat liver tissues with positive reaction

	Indexes			
	
	CD14 activation		Endotoxin	
**Time**	**Sham**	**Ligation**	**Sham**	**Ligation**

	N (%)*	N (%)	N (%)	N (%)
3 h (n = 6)	1 (16.7)	4 (66.7)	6 (100)	6 (100)
6 h(n = 6)	1 (16.7)	4 (66.7)	0	4 (66.7)
12 h (n = 6)	2 (33.3)	1 (16.7)	1 (16.7)	3(50)
24 h (n = 6)	1 (16.7)	0	0	1 (16.7)
3 d (n = 6)	0	2 (33.3)	1 (16.7)	1 (16.7)
7 d (n = 6)	0	2 (33.3)	0	5 (83.3)
14 d (n = 6)	1 (16.7)	5 (83.3)	1 (16.7)	6 (100)
21 d (n = 6)	1 (16.7)	6 (100)	0	6 (100)

*Immunohistochemical CD14 and endotoxin staining in the liver tissues of rat among

sham and common bile duct ligation group. The positive cells were >10% as positive.

**Figure 10:**
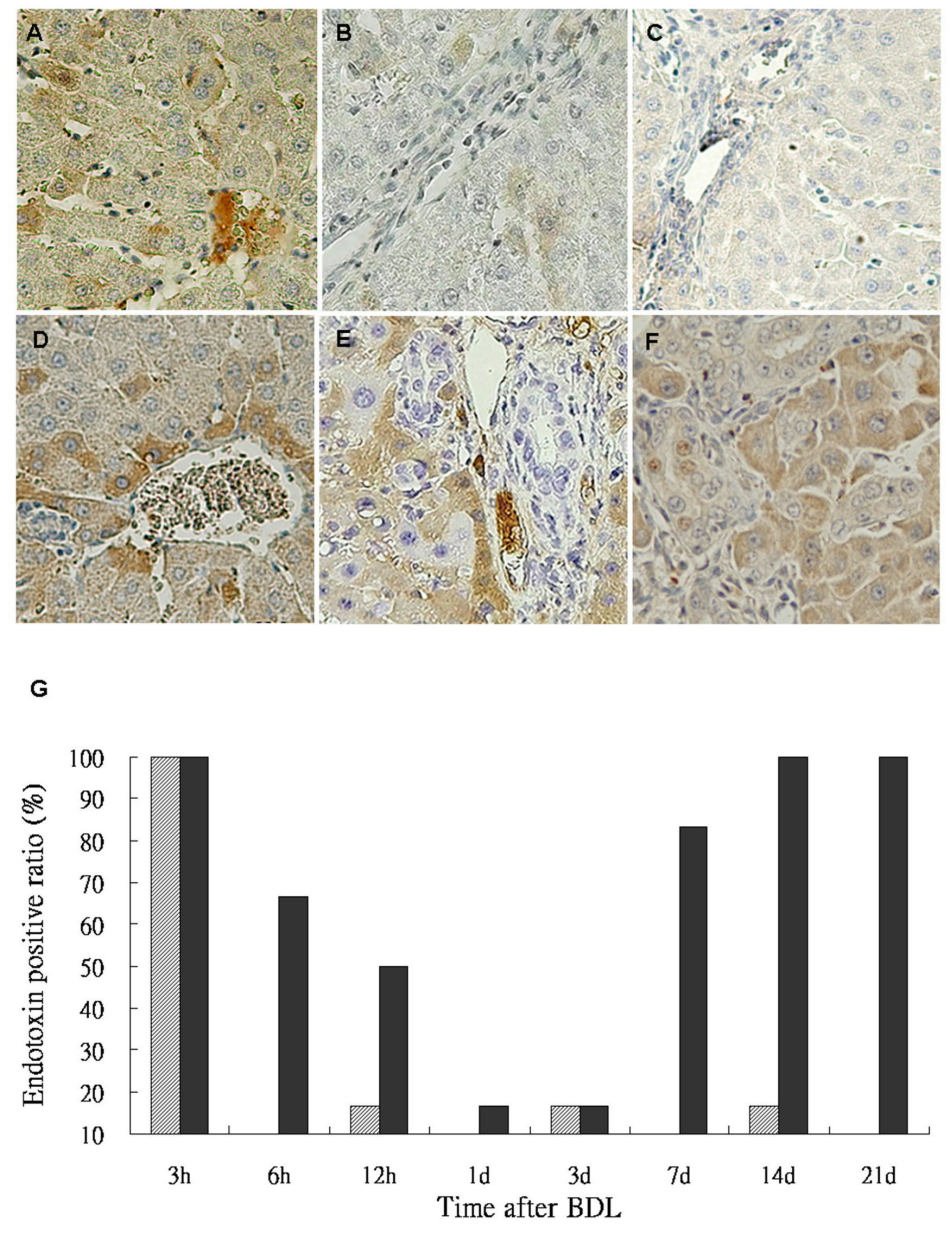
**Endotoxin staining in the liver tissues of rats**. Immunohistochemical stain of the liver sections using a monoclonal antibody against lipid A shows positive staining in the Kupffer cells, hepatocytes, and the sinusoidal endothelial cells at 3 h (A), 1 week r(B) and 3 week (C) after sham-operated; and at 3 h (D), 1 week (E) and 3 week (F) after BDL. Tissues were counterstained with hematoxylin. Original magnification: × 200. Statistical analyses of the immunohistochemical score of >10% endotoxin in liver sections of the sham (pale bar) and BDL groups (black bar) (G).

Hepatic endotoxin levels were higher in the BDL rats (Figure 10D-F) compared with the sham-operated group (Figure 10A-C) by immunohistochemical staining. Significantly higher endotoxin accumulation was observed in hepatocytes following BDL. Based on the extent and intensity of anti-lipid A stain, a semiquantitative method was used to calculate the ratio with the positive area over 10% in liver sections. As shown in Table 2 and Figure 10G, endotoxin was detected in liver tissues at 3 h in BDL and sham-operated rats. Like CD14 activation in the BDL group, endotoxin accumulation was returned to baseline levels by 24 h and then increased again after 7 days post ligation.

## Discussion

Our results demonstrated for the first time the expression profile of sCD14 in patients with BA and found significantly higher CD14 mRNA and protein levels in early-stage BA relative to late-stage BA and CC. However, hepatic endotoxin levels remained very high, despite a significant increase in plasma endotoxin levels in patients with BA compared with control patients. The liver is thought to be involved in the systemic clearance and detoxication of endotoxin, and Kupffer cells and hepatocytes both contribute to clearing endotoxin via different recognition systems [24,25]. The production of sCD14 and LPS binding protein by hepatocytes could provide a powerful mechanism by which the liver carries out its function of clearing endotoxin from the blood stream [26,27].

CD14 expression in the liver increased in many types of liver disease, including alcohol and cholestatic liver injuries in rodents [17,28-30]. Immunohistochemical analysis performed in this study showed higher CD14 expression in Kupffer cells and sinusoidal endothelial cells in early-stage BA relative to late-stage BA. When the phagocytic function of Kupffer cells is impaired in cholestasis, portal derived endotoxin may accumulate in the liver and spill over into the peripheral circulation from the intestine [31-33]^. ^It is suspected that high expression of CD14 in Kupffer cells and sinusoidal endothelial cells may imply a response of these cells to cholestatic liver injury or to increased endotoxin as a result of cholestasis. However, the localization of CD14 mRNA was mainly observed in hepatocytes and bile duct epithelial cells. Therefore, we cannot rule out the possibility that CD14 production by hepatocytes and bile duct epithelial cells during cholestasis and its subsequent transportation to the Kupffer cells and sinusoidal endothelial cells produces an unknown downstream effect. Our results provided evidence that BA does not exempt from endotoxin accumulation in the peripheral circulation and liver, just like other cholestatic disorders. sCD14 have been demonstrated direct secretion by hepatocytes during early-stage of BA to enhance endotoxin clearance [15,34]. During cholestasis, the accumulation of endotoxin may induce hepatocyte injury and impair CD14 synthesis during late-stage BA.

Although studies of liver injury should take into account the relative contributions of CD14 in Kupffer cells and hepatocytes, few reports document the proportional change of mCD14 and sCD14 in the liver and the consequent pathogenetic effects on cholestatic diseases [16,29]. The balance between activation and inhibition of endotoxin responses by sCD14 depends on its concentration [35,36]. At a higher physiological concentration, sCD14 can compete with mCD14 and inhibit LPS activation of CD14-positive cells [35,36]. It is likely that increased plasma levels of sCD14 during early-stage BA observed in this study implies a protective mechanism in the liver that guards against increased endotoxin due to cholestasis. Conversely, decreased sCD14 in late-stage BA, without a concomitant decrease of endotoxin in the liver and blood, may indicate a loss of protection against CD14-mediated LPS activation in Kupffer cells that propagates inflammatory reactions and fibrogenesis, resulting in irreversible liver injury and end-stage liver cirrhosis in patients with BA [10,16,35].

In an animal model system injected intraperitoneally with LPS, the initial and rapid induction of CD14 expression in myeloid cells is followed by a second, slower response in epithelial cells, which peaks at 8-16 h [37]. This epithelial cell response appears to require higher concentrations of LPS induction and is dependent on TNF-α to promote synthesis of CD14 [38]. Apart from its apparent role as an LPS receptor that mediates activation of myeloid cells, CD14 also appears to serve as an opsonic receptor for engulfment by phagocytes, resulting in clearance of LPS. Liver is the main clearance organ for intravenously injected LPS, and this is mediated by Kupffer cells, sinusoidal cells, granulocytes, and hepatocytes in rats [39]. There is evidence to suggest that LPS may be cleared from the liver via the bile canalicular system into the gut. Indeed, 3 h after LPS injection, bile samples taken from the gall bladder of rabbits contained substantial amounts of LPS, equivalent to that found in the plasma [40]. In our BDL rat model, we confirmed that endotoxemia and hepatocyte CD14 production occurred after ligation. CD14 was expressed in an LPS-inducible manner in Kupffer cells, neutrophils, hepatocytes, and bile duct epithelium, suggesting a possible role for CD14 in hepatocytes during the uptake and clearance of LPS from the circulation. However the endotoxin levels in liver tissues were still high due to cholestasis, and CD14 production was increased again at 7 days after ligation.

Although sCD14 has been observed in normal human serum and is increased in sera from septic patients [14,41]. The origin of sCD14 has yet to be determined. It has been assumed that sCD14 is derived from the membrane bound form present on myeloid cells either by phospholipase cleavage of the GPI anchor or by protease digestion [14]. However, patients with paroxysmal nocturnal hemoglobinuria have normal sCD14 levels in their sera, although monocytes from these patients do not express CD14 on their surface [42]. Our observation that the CD14 mRNA is detected in hepatocytes raises the possibility that sCD14 may also originate from these cells. Recent studies have shown that the CD14 antigen is expressed in many types of cells and tissues [37,43,44]. Some reports suggest that sCD14 behaves like other acute-phase proteins [14]. Hepatocytes are the major source of most acute-phase proteins; therefore, hepatocytes might be expected to express CD14, which is upregulated during endotoxemia induced by cholestasis [10,16,39]. These results are in agreement with other previous reports that demonstrated the synthesis and expression of CD14 are markedly upregulated by LPS during endotoxemia induced by cholestasis [3,8,43]. In the liver, besides hepatocytes, nonparenchymal cells such as Kupffer cells, endothelial cells, neutrophils, and other cells can also express the CD14 mRNA and synthesize the CD14 protein [21,22,26], but the fact that both isolated hepatocytes and hepatic tissues express the CD14 protein and its mRNA indicate that the nonparenchymal cells could hardly have any effect on such expression in liver tissues. Although we do not provide direct evidence here that sCD14 in the plasma originates from hepatocytes during endotoxemia, our results showed that there was the possibility that the liver is an important source of sCD14 during endotoxemia. Pan *et al *[21]. found that the liver is one of the major organs involved in the production of sCD14. Liu *et al *[26] also reported that CD14 transcription rates are significantly increased in hepatocytes from LPS-treated rats, indicating that the upregulation of CD14 mRNA levels observed in rat hepatocytes after LPS treatment is dependent, in part, on increased transcription. Their observations also support the idea that sCD14 could be an acute-phase protein and hepatocytes might be a source of circulating sCD14 production. Our data indicated that hepatocytes from BDL rats expressed higher amounts of CD14 mRNA and protein and may have released more sCD14 for promoting endotoxin clearance.

## Conclusions

In conclusion, our *in vivo *data indicated the liver as a main source of sCD14 production during endotoxemia. However, the significantly decreased sCD14 expression in late-stage BA without a concomitant decrease in plasma endotoxin levels suggested that the pathogenetic mechanism underlying CD14-mediated liver injury during BA is still unresolved.

## Abbreviations

(BA): biliary atresia; (BDL): bile duct ligation; (KP): Kasai procedure; (qRT-PCR): real-time quantitative reverse-transcription polymerase chain reaction

## Competing interests

The authors declare that they have no competing interests.

## Authors' contributions

MHC performed the experiments and drafted the manuscript. JHC designed the experiment and clinical specimen collection. HLE was the pathologist and evaluated the histopathology of the cases. CMC participated in experiment performance and technical support. CHW helped analyze the data. CLC coordinated the study and drafted the manuscript. TML provided scientific advice, discussions of data and submitted the manuscript. All authors read and approved the final manuscript.
